# The reference genome sequence of *Artemisia argyi* provides insights into secondary metabolism biosynthesis

**DOI:** 10.3389/fpls.2024.1406592

**Published:** 2024-06-28

**Authors:** Xinqiang Gao, Qiang Ma, Xiaomeng Zhang, Xingyun Wang, Nuohan Wang, Yupeng Cui, Shuyan Li, Shengming Ma, Hong Wang, Kunpeng Zhang

**Affiliations:** ^1^ College of Biology and Food Engineering, Anyang Institute of Technology, Anyang, China; ^2^ Henan Artemisia Argyi Medical Research Center, Anyang, China

**Keywords:** *Artemisia argyi*, Chinese traditional medicine, reference genome, MeJA, transcriptome

## Abstract

*Artemisia argyi*, a perennial herb of the genus *Artemisia* in the family Asteraceae, holds significant importance in Chinese traditional medicine, referred to as “Aicao”. Here, we report a high-quality reference genome of *Artemisia argyi* L. cv. beiai, with a genome size up to 4.15 Gb and a contig N50 of 508.96 Kb, produced with third-generation Nanopore sequencing technology. We predicted 147,248 protein-coding genes, with approximately 68.86% of the assembled sequences comprising repetitive elements, primarily long terminal repeat retrotransposons(LTRs). Comparative genomics analysis shows that *A. argyi* has the highest number of specific gene families with 5121, and much more families with four or more members than the other 6 plant species, which is consistent with its more expanded gene families and fewer contracted gene families. Furthermore, through transcriptome sequencing of *A. argyi* in response to exogenous MeJA treatment, we have elucidated acquired regulatory insights into MeJA’s impact on the phenylpropanoid, flavonoid, and terpenoid biosynthesis pathways of *A. argyi*. The whole-genome information obtained in this study serves as a valuable resource for delving deeper into the cultivation and molecular breeding of *A. argyi*. Moreover, it holds promise for enhancing genome assemblies across other members of the Asteraceae family. The identification of key genes establishes a solid groundwork for developing new varieties of *Artemisia* with elevated concentrations of active compounds.

## Introduction

The Chinese traditional medicine “Aicao” is mostly prepared from the dried leaf of *Artemisia argyi* Levl. et Van, a perennial herb that belongs to the Asteraceae species (Chinese Pharmacopoeia Commission, 2010). Generally, “Aicao” is harvested twice in June and October a year and is obtained by reaping the leaf and stem before drying. *A. argyi* has played an important role in clinical treatment in China for thousands of years because of its extensive pharmacological effects, such as hemostasis, antiphlogistic, antitussive, pain relief, tocolysis, antiallergic and so on. At the same time, the dried leaf of *A. argyi* is also the main material of famous international moxibustion therapy, with remarkable curative effect, such as menstruation-related symptoms, eczema, diarrhoea and tuberculosis (Sun et al., 2019). The brilliant work of the Ming Dynasty, Compendium of Materia Medica (A.D. 1552–1578), already described *A. argyi*. To date, the chemical components isolated from *A. argyi* include mainly terpenoids, flavonoids and phenylpropanoids (Yoshikawa et al., 1996; Zhang et al., 2013; Han et al., 2017). Several of these characteristic components have been investigated by modern science to confer health benefits, such as antioxidant, anti-tumor, anti-inflammatory, anticoagulant, antibiosis, as well as neuroprotection and immunomodulation among others (Seo et al., 2003; Guan et al., 2006; Bao et al., 2013; Zeng et al., 2014; Lv et al., 2017; Xiang et al., 2018; Guan et al., 2019; Xiao et al., 2019; Tseng et al., 2020; Zhang et al., 2020). As a rare and valuable medicinal material in China, *A. argyi* is also used in food and daily chemical products. It is in widespread use, with total annual output values exceeding 10 billion RMB.

Despite the commercial interest and increasing demand for *A. argyi*, improvements through breeding have been very limited. The absence of genome information has limited any improvement in productivity through genetic selection and limited understanding of how its secondary metabolites biosynthesis. Whole-genome sequencing has become a practical strategy to identify metabolic pathways for natural product biosynthesis (Liu et al., 2017; Mochida et al., 2017). Up to now, many Chinese herbal medicines have completed genome sequencing, such as *Artemisia annua*, *Salvia miltiorrhiza*, *Scutellaria baicalensis*, *Papaver somniferum*, *Taxus chinensis*, which provide strong support for the development of related industries and scientific research (Xu et al., 2016; Li et al., 2018; Qian et al., 2018; Zhao et al., 2019; Cheng et al., 2021). In addition, a large number of studies have shown that plant hormone methyl jasmonate (MeJA) acts as an effective elicitor for natural production of secondary metabolites across the plant kingdom, including some traditional Chinese medicinal plants, like *Artemisia annua*, *Taxus chinensis*, *Salvia miltiorrhiza*, and *Catharanthus roseus* (Li et al., 2012; Liu et al., 2016; Shi et al., 2016; Hao et al., 2017). To elucidate the regulation mechanism of MeJA on the biosynthesis of terpenoids and other secondary metabolites by using the whole genome and transcriptome sequencing, is of great significance for the effective production of secondary metabolites of *A. argyi* by synthetic biological methods or genetic improvement.

Here, we report a high-quality genome assembly of *A. argyi* by third-generation Nanopore sequencing technology. In total, 4.15 Gb genome were assembled, with a contig N50 of 508.96 Kb, and 147,248 genes were predicted in the *A. argyi* genome, 68.86% of which were repetitive elements. We performed phylogenetic and comparative genomic analyses to investigate phylogenetic diverge, and expansion/contraction of gene families. In addition, we also carried out the transcriptome sequencing of *A. argyi* responding to exogenous MeJA treatment to obtain the regulatory information of MeJA on phenylpropanoid, flavonoid, and terpenoid biosynthesis pathways of *A. argyi*. Our study not only sheds light on the evolution of specific terpenoid biosynthetic pathways in *A. argyi* but also establishes the genome information as a valuable resource for further investigation into the growth characteristics, cultivation, and molecular breeding of *A. argyi*.

## Materials and methods

### Sample collection and DNA preparation

Mature leaves of *Artemisia argyi* L. cv. beiai were collected from a single plant maintained in Tangyin Bian Que Temple (Anyang, Henan Province, China). The leaves were frozen in liquid nitrogen and stored at -80°C until DNA extraction. Total genomic DNA was extracted from the leaf tissues following the CTAB protocol (Murray and Thompson, 1980).

### Genome size estimation

The genome size of *A. argyi* was estimated using K-mer (k = 21) analysis (Marçais and Kingsford, 2011). Briefly, genomic DNA was extracted and sonicated to a fragment size of 350 bp, then 5 libraries was built followed by terminal repairing, poly A and adaptor adding, target fragment selection and PCR processes. The resulted library was qualified using Agilent 2100 and qPCR methods. Then the library was fixed on the microarray by the bridge PCR before it was sequenced on Illumina sequencing platform. The 21-mer frequencies were generated using 215.77 Gb high-quality PE reads. A k-mer is an oligonucleotide sequence of length k extracted from the sliding windows of sequencing data. Under the premise of a uniform distribution of sequencing reads, the following formula is obtained:


Genomic size = total number of bases / average sequencing depth

= total kmer / median kmer depth

A k-mer map of k = 21 was constructed using the 350-bp library data for the evaluation of genome size. The main peak corresponding to the k-mer depth was 45, which was the average k-mer depth. A sequence in which the k-mer depth appeared to be more than twice the depth of the main peak (depth value, 90) was considered a repeat sequence. A k-mer depth was half of the main peak (depth value, 22), indicating that the sequence was heterozygous.

A second determination of the genome size was based on flow cytometry (Zhang et al., 2012). Nuclear DNA was isolated from fresh leaf tissue using a Partec CyStain UV Precise P kit, and flow cytometry (Partec CyFlow Space) was applied to estimate the nuclear DNA content. The reference standard was *Triticum aestivum* L. (15.5 Gb). The genome size of *A. argyi* was calculated as the ratio between the sample and standard peaks multiplied by the genome size of the standard.

### Nanopore sequencing

Nanopore sequencing 2 μg of gDNA was repaired using NEB Next FFPE DNA Repair Mix kit (M6630, USA) and subsequently processed using the ONT Template prep kit (SQK-LSK109, UK) according to the manufacturer’s instructions. The large segments library was premixed with loading beads and then pipetted into a previously used and washed R9 flow cell. The library was sequenced on the ONT PromethION platform with Corresponding R9 cell and ONT sequencing reagents kit (EXP-FLP001.PRO.6, UK) according to the manufacturer’s instructions.

### *De novo* genome assembly of nanopore reads

*De novo* genome assembly was performed by combination strategies: First, the nanopore three generation data were corrected by the correction function in Canu (https://github.com/marbl/canu, v1.5) (Sergey et al., 2017). Second, the corrected reads were assembled by WTDBG2 (https://github.com/ruanjue/wtdbg2). Third, Racon (Racon, RRID: SCR 017642) was used to calibrate the draft genome with third-generation data through 3 rounds (Vaser et al., 2017). Fourth, Pilon v1.21 (Pilon, RRID: SCR 014731) was used to calibrate the draft genome with second-generation data through 3 rounds (Walker et al., 2014).

The integrity of the assembled genome was assessed. First, BWA v0.7.15 (BWA, RRID: SCR 010910) was used to compare the two generation data with the reference genome (Durbin, 2009). Second, CEGMA v2.5 (CEGMA, RRID: SCR 015055) was used to assess the integrity of 458 conserved CEGs and 248 highly conserved CEGs (Parra et al., 2007). Third, the BUSCO database v4.0 (BUSCO, RRID: SCR 015008) was used to assess the completeness of gene regions, which contained 1,614 conserved core genes (Simão et al., 2015).

### Repeat sequences, non-coding RNA and pseudogene analysis

Transposon element (TE) and tandem repeat were annotated by the following workflows. TE were identified by a combination of homology-based and *de novo* approaches. We first customized a *de novo* repeat library of the genome using RepeatModeler2 (v2.0.1), which can automatically execute two *de novo* repeat finding programs, including RECON (v1.08) and RepeatScout (Bao, 2002; Price et al., 2005; Flynn et al., 2020). Then full-length long terminal repeat retrotransposons (fl-LTR-RTs) were identified using both LTRharvest (-minlenltr 100 -maxlenltr 40000 -mintsd 4 -maxtsd 6 -motif TGCA -motifmis 1 -similar 85 -vic 10 -seed 20 -seqidsyes) and LTR_finder (-D 40000 -d 100 -L 9000 -I 50 -p 20 -C -M 0.9). The high-quality intact fl-LTR-RTs and non-redundant LTR library were then produced by LTR_retriever (Zhao and Hao, 2007; Ellinghaus et al., 2008; Ou and Ning, 2018). Non-redundant species-specific TE library was constructed by combining the *de novo* TE sequences library above with the known Repbase (v19.06), REXdb (V3.0) and Dfam (v3.2) database (Jurka et al., 2005; Travis et al., 2013; Neumann et al., 2019). Final TE sequences in the *A. argyi* genome were identified and classified by homology search against the library using RepeatMasker (v4.10) (Tarailo-Graovac and Chen, 2009). Tandem repeats were annotated by Tandem Repeats Finder (TRF, v409) and MIcroSAtellite identification tool (MISA v2.1) (Benson, 1999; Sebastian et al., 2017).

Non-coding RNAs are usually divided into several groups, including miRNA, rRNA, tRNA, snoRNA and snRNA. The tRNAscan-SE (v1.3.1) was used to predict tRNA with eukaryote parameters (Lowe and Eddy, 1997). Identification of the rRNA genes was conducted by Rfam (v12.0) and barrnap(v 0.9) (Griffiths-Jones et al., 2005; Loman, 2017). The miRNA was identified by searching miRBase (v21) databases (Griffiths-Jones et al., 2006). The snoRNA and snRNA genes were predicted using INFERNAL1.1 against the Rfam (v12.0) database (Griffiths-Jones et al., 2005; Nawrocki and Eddy, 2013).

Pseudogenes usually have similar sequences to functional genes, but may have lost their biological function because of some genetic mutations, such as insertion and deletion. The GenBlastA (v1.0.4) program was used to scan the whole genomes after masking predicted functional genes (Rong et al., 2009). Putative candidates were then analyzed by searching for non-mature mutations and frame-shift mutations using GeneWise (v2.4.1) (Birney, 2004). Motif and domain annotation were predicted by InterProScan (5.34–73.0) (Philip et al., 2014).

### Protein-coding gene prediction and functional annotation

We integrated three approaches, namely, *de novo* prediction, homology search and transcript-based assembly, to annotate protein-coding genes in the genome. The *de novo* gene models were predicted using two ab initio gene-prediction software tools, Augustus (v2.4) and SNAP(2006–07-28) (Ian, 2004; Baertsch and Haussler, 2008). For the homolog-based approach, GeMoMa (v1.7) software was performed by using reference gene model from the *A. annua*, *A. thaliana*, *C. nankingense* and *H. annuus* species (Jens et al., 2016). For the transcript-based prediction, RNA-sequencing data were mapped to the reference genome using Hisat (v2.0.4) and assembled by Stringtie (v1.2.3) (Kim et al., 2015; Pertea et al., 2015). GeneMarkS-T (v5.1) were used to predict genes based on the assembled transcripts (Shiyuyun et al., 2015). The PASA (v2.0.2) software was used to predict genes based on the unigenes (and full-length transcripts from the ONT sequencing) assembled by Trinity (v2.11) (Haas et al., 2003; Grabherr et al., 2013). Gene models from these different approaches were combined using the EVM software (v1.1.1) and updated by PASA (Haas et al., 2008). The final gene models were annotated by searching the GenBank Non-Redundant (NR, 20200921), EggNOG (5.0), TrEMBL (202005), Pfam (33.1), SwissProt (202005), eukaryotic orthologous groups (KOG, 20110125), gene ontology (GO, 20200615) and Kyoto Encyclopedia of Genes and Genomes (KEGG, 20191220) databases. All parameters were set as 1E^-5^ (Tatusov, 2001; Boeckmann, 2003; Conesa et al., 2005; Finn, 2006; Aron et al., 2011; Minoru et al., 2016; Jaime et al., 2018).

### Gene families cluster analysis

The available protein sequence sets were collected from 7 sequenced plant species: *A. argyi*, *A. annua*, *C. nankingens*e, *C. canephora*, *M. micrantha*, *L. sativa* and *V. vinifera*. Orthofinder (v2.4) software (the alignment method used was diamond, and the alignment e-value was 0.001) was used to classify the protein sequences, and Panther V15 database was used to annotate the obtained gene families (Mi et al., 2018; Emms and Kelly, 2019). Go and KEGG enrichment analysis were performed for the *A. argyi*-specific gene families by clusterProfile (v3.14.0) (Yu et al., 2012).

### Phylogenetic tree construction

383 single-copy genes were used for phylogenetic tree construction with 6 other sequenced plant genomes by IQ-TREE (v1.6.11) (Lam-Tung et al., 2015). The concrete methods are as follows. MAFFT (v7.205) was used to compare each single copy gene family sequence (parameter: -localpair -maxiterate 1000) (Katoh et al., 2009). The protein alignment was transformed into codon alignment by PAL2NAL (V14) program (Mikita et al., 2006). Gblocks (parameter: b5 = h) was used to remove regions with poor sequence alignment or large differences (Gerard and Jose, 2007). All the gene family sequences were connected end-to-end to obtain a supergene. IQFinder’s built-in model detection tool ModelFinder was used for model detection, and the best model obtained was GTR + F + I + G4 (Kalyaanamoorthy et al., 2017). This best model was then used to construct an evolutionary tree using the maximum likelihood (ML) method, with the number of bootstrap replicates set to 1,000. MCMCTREE, a software package that comes with PAML (v4.9e), was used to calculate divergence times of *A. argyi* from the other plants (Yang, 2007; Puttick, 2019). In addition, the divergence times of *V. vinifera* Vs *C. canephora* (110–124 Mya), *C. canephora* Vs *L. sativa* (93–107 Mya), and *L. sativa* Vs *A. annua* (32–41 Mya) from TimeTree (http://www.timetree.org/) were used for fossil calibration.

### Gene family expansion and contraction analysis

CAFE (Computational Analysis of gene Family Evolution) software was used to analyze divergence times and gene family expansion and contraction (Han et al., 2013). The results of evolutionary tree and gene family clustering were used to estimate the number of gene families of the ancestors in each phylogenetic tree branch, thereby predicting gene family contraction and expansion. The criterion for defining significant expansion or contraction was a *P*-value < 0.05.

### Positive selection analysis

The CodeML module in PAML was used for positive selection analysis. Single-copy genes of *A. annua*, *A. argyi*, *C. nankingense*, *L. sativa* and *M. micrantha* were obtained, and the protein sequence of each gene family was compared using MAFFT (parameter: localpair -maxiterate 1000). The “chi2” program in the PAML program was used to perform likelihood ratio tests on Model A (assuming that the foreground branch ω was in a positive choice, i.e., ω > 1) and the null model (meaning that the ω value of any site was not allowed to be >1), with significance assessed at *P* < 0.01. The Bayesian method (BEB, Bayes empirical Bayes method) was used to obtain positive selection sites (greater than 0.95 is usually considered significantly positively selected sites), and the genes receiving significant positive selection were ultimately obtained.

### MeJA treatment and transcriptome sequencing

For MeJA treatment, 50-day-old *A. argyi* L. cv. beiai seedlings were sprayed with 100 μM MeJA (Sigma-Aldrich, USA). For the mock treatment, seedlings were sprayed with 0.1% ethanol. Seedling samples were collected at 0, 1, 3, 6, 12 and 24 hours after treatment. The treatments were carried out with three biological replicates. At each treatment time, flesh leaves were collected and frozen in liquid nitrogen and stored at -80°C for further use. The main RNA-seq steps were as follows: (a) total RNA sample detection; (b) library construction; (c) library inspection; (d) sequencing and bioinformatics.

Raw data (raw reads) of fastq format were firstly processed through in-house perl scripts. In this step, clean data(clean reads) were obtained by removing reads containing adapter, reads containing ploy-N and low quality reads from raw data. At the same time, Q20, Q30, GC-content and sequence duplication level of the clean data were calculated. All the downstream analyses were based on clean data with high quality. These clean reads were then mapped to the reference genome sequence. Only reads with a perfect match or one mismatch were further analyzed and annotated based on the reference genome. Hisat2 tools soft were used to map with reference genome (Kim et al., 2015). Quantification of gene expression levels were estimated by fragments per kilobase of transcript per million fragments mapped. Differential expression analysis of two conditions/groups was performed using the DESeq2 (Love et al., 2014). DESeq2 provide statistical routines for determining differential expression in digital gene expression data using a model based on the negative binomial distribution. The resulting *P* values were adjusted using the Benjamini and Hochberg’s approach for controlling the false discovery rate. Genes with an adjusted *P*-value < 0.05 found by DESeq2 were assigned as differentially expressed. Genes with an adjusted *P*-value < 0.05 and Fold change ≥1.5 found by DESeq2 were assigned as differentially expressed.

## Results

### Genome sequencing, assembly and annotation

**Figure 1:**
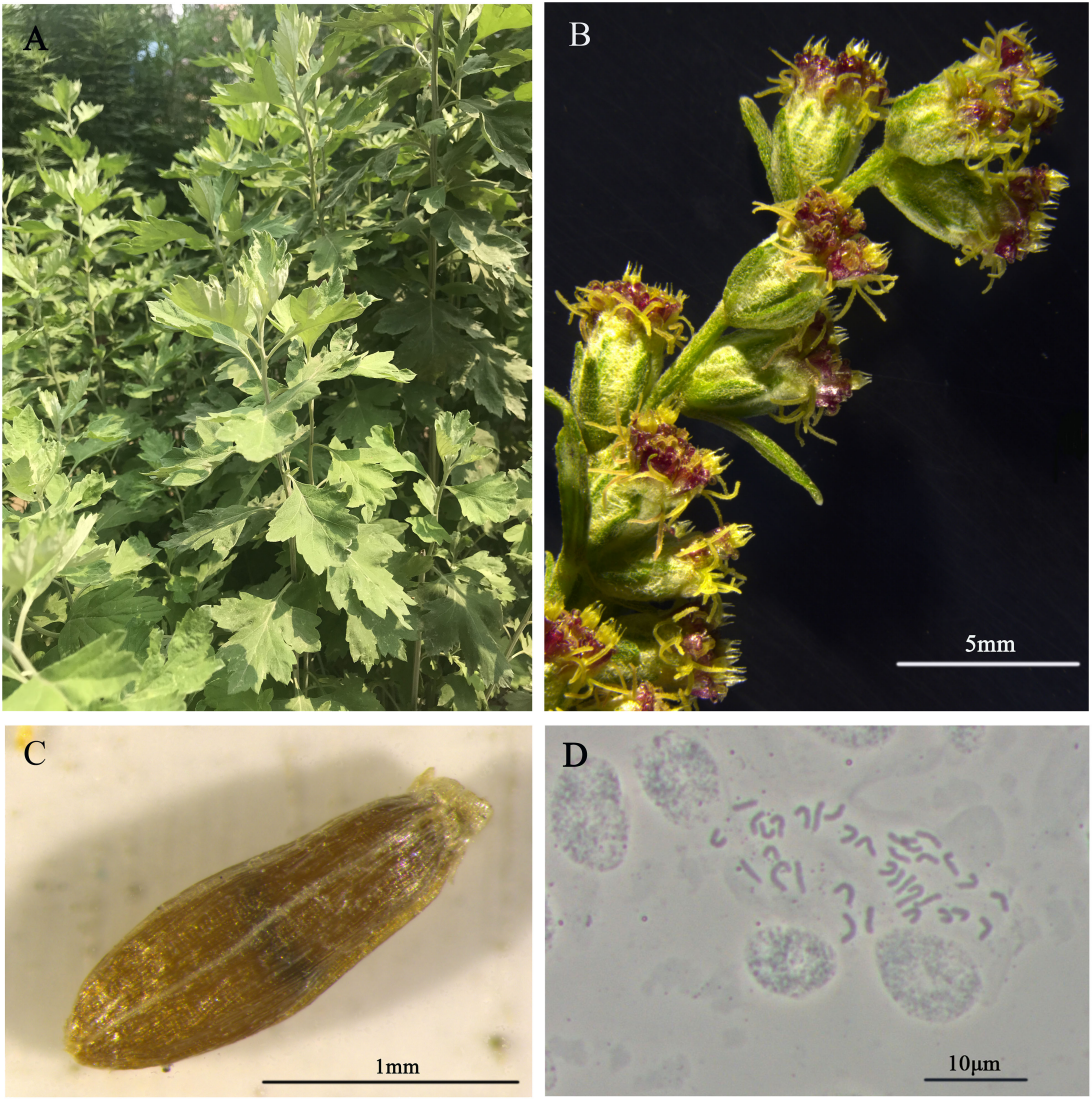
Morphological characters of the *A. argyi*. Mature plants in field **(A)**, flowers **(B)**, seed **(C)** and karyotype **(D)** are shown.

**Figure 2:**
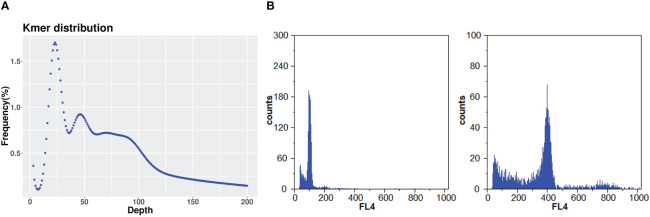
Estimation of *A. argyi* genome size. **(A)** K-mer analysis. The 21-mer frequency distribution derived from the sequencing reads was plotted. **(B)** Flow cytometer analysis. The left panel showing the *A. argyi* at 100.53, while the right panel indicating *Triticum aestivum* L. at 399.08 with 15.5 Gb.

The DNA for genome sequencing of *A. argyi* came from a single plant maintained in Tangyin Bian Que Temple, Anyang, Henan Province, China (**Figures 1A–C**). The somatic cells of A. argyi contained 34 chromosomes by the cytological observation method (**Figure 1D**). Due to the wide variety of *A. argyi*, it was necessary to obtain information on the genome size and heterozygosity of the *A. argyi* genome. Five 350 bp libraries were constructed using genomic DNA from leaf samples, and 215.77 Gb of highquality data was sequenced and filtered, representing ~54×. The sequencing data of Q20 ratio and Q30 ratio was all above 97.02% and 92.09%, respectively (**Supplementary Table 1**). K-mer analysis (**Figure 2A**) of this data set indicated that the *A. argyi* genome has a genome size of ~3.99 Gb and a level of heterozygosity (0.58%). Flow cytometry analysis (**Figure 2B**) indicated that the *A. argyi* genome size was ~3.92 Gb, which was slightly smaller than the estimate obtained by K-mer analysis.

The *A. argyi* genome was sequenced using the third-generation sequencing technology of Nanopore. After further filtering out the adapters, low-quality reads, and short fragments (length < 2000 bp), we obtained 18,218,868 clean reads for a total of 477.82 Gb of high-quality sequences (**Supplementary Table 2**), representing ~115× coverage of this *A. argyi* genome. Notably, the average length of the reads was 41,642 bp, and most of the clean reads were distributed in the range of 20,000 - 50,000 bp (**Supplementary Table 3**).

The sequenced ONT clean data were then assembled into the final genome using different assembly combination strategies, namely, Canu, Wtdgb, Racon and Pilon, according to the standard protocols for each strategy. The final genome assembly was 4.15 Gb in size, very close to the predicted size of 3.99 Gb and 3.92Gb based on K-mer analysis and flow cytometry, respectively (**Figure 2**), and it consisted of 17,220 contigs, with a contig N50 of 508,957 bp and a contig N90 of 132,683 bp (**Supplementary Table 4**). The GC content of the assembled *A. argyi* genome was 35.25% (**Table 1**).

**Table 1:** Statistics of *A. argyi* genome sequencing, assembly and annotation.

Category	Data
Genome size (Gb)	4.15
Number of contigs (≥1 kb)	17,220
N50 contig length (kb)	508,957
Largest contig (kb)	3,649,437
GC content (%)	35.25
Number of putative genes	147,248
Gene length (Mb)	459.815
Mean gene length (bp)	3,122.73
Total exon length (Mb)	176.371
Mean exon length (bp)	1,197.78
Total intron length (Mb)	283.444
Mean intron length (bp)	1,924.94

The quality of the assembly was further assessed by three independent methods. First, the second-generation high-throughput sequencing data were aligned to the genome sequence using BWA software, the results indicated that more than 99.15% of the sequences could be mapped to the assembled genome, and the properly mapped (paired-end reads mapped to the genome with a distance consistent with the length distribution of the sequenced fragments) read rate was 83.94% (**Supplementary Table 5**). Second, 444 (96.94%) conserved genes and 236 (95.16%) highly conserved genes were identified in the 458 eukaryotic conserved sequences and 248 highly eukaryotic conserved sequences using CEGMA, respectively (**Supplementary Table 6**). Third, BUSCO was used to search the conserved plant genes (1614 conserved plant genes in the database) in the assembled *A. argyi* genome, and 1465 genes, accounting for 90.77% of the total genes in the database, were identified (**Supplementary Table 7**). Thus, all of these results support the conclusion that this assembled *A. argyi* genome is of high quality.

A total of 147,248 putative genes were predicted by combining the *de novo*, homology-based and transcriptome-based predictions, with an average gene length of 3,122 bp and 4.45 exons per gene; 77.55% of these genes shared homology with known genes, and 97.56% of these genes were functionally annotated (**Table 1**; **Supplementary Tables 8**, **9**; **Supplementary Figure 1**). Among the genomes in the Asteraceae family, the number of genes in the *A. argyi* genome was much more than the numbers estimated for the *A. annua* (63,226 genes) and *C. nankingense* (56,870 genes) genomes (**Supplementary Table 10**). A total of 5,912 noncoding RNAs, including 65 rRNAs, 4,248 tRNAs, 336 miRNAs, 708 snRNA and 555 snoRNA, were predicted by different strategies (**Supplementary Table 11**). In addition, 4,050 pseudogenes were predicted in the *A. argyi* genome (**Supplementary Table 12**). Motif and domain annotation analysis based on the amino acid sequences of 147,248 predicted genes indicated a total of 3,342 motifs and 108,572 domains (**Supplementary Table 13**).

### Repetitive sequences and recent bursts of LTR retrotransposons

Through a combination of approaches, we annotated 68.86% (~2.86 Gb) of the assembly as repetitive elements (**Supplementary Table 14**). The long terminal repeat retrotransposons (LTRs) were the most abundant (38.64%), and most LTRs were LTR/Gypsy elements, which occupied 21.37% of the genome, followed by the LTR/Copia repeats (17.27%, **Supplementary Table 14**). Besides the main groups of LTR elements, 4.23% of the genome was annotated as DNA transposons, whereas the remainder was either assigned to other repeat families or could not be assigned (**Supplementary Table 14**). We also predicted 216.77 Mb tandem repetitive sequence (TRS), represented 5.22% of the genome assembly (**Supplementary Table 15**).

We further identified 658,642 and 627,908 intact Gypsy and Copia retrotransposons (**Supplementary Table 14**), respectively. The predicted time of the LTR retrotransposon burst in *A. argyi* was ~1.18 Mya (**Supplementary Figure 2**), which was comparable with that recently reported in *C. nankingense* (~1.46 Mya), with a large genome (~2.5 Gb) (Song et al., 2018). Thus, these data suggest that recent insertions of LTR elements may have contributed to the increase in *A. argyi* genome size.

### Comparative genomic and genome evolutionary analysis

**Figure 3:**
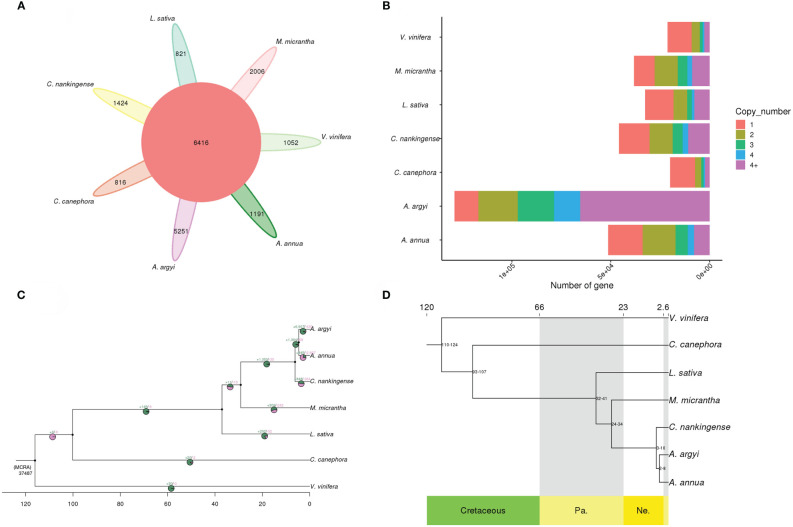
Gene families and phylogenetic analysis. **(A)** Petal diagram of the gene families of 7 species. The middle circle is the number of gene families shared by all species, and the number of gene families unique to each species is on the side. **(B)** Copy number distribution of the gene families of 7 species. **(C)** CAFE-based estimates of gene family expansions and contractions. The numbers after “+” and “−” represent the numbers of expanded and contracted gene families, respectively. The green in the pie chart represents gene family expansion, and the pink represents contraction. **(D)** Phylogenetic tree of *A. argyi* and 6 other species. At the bottom of the tree is geological time (prefix), and at the top of the tree is absolute age (measured in Mya). The tree is rooted with *V. vinifera* as the outgroup. Cretaceous, Pa. Paleogene, Ne. Neogene.

A gene family cluster analysis of the complete gene sets of *A. annua*, *A. argyi*, *C. canephora*, *C. nankingense*, *L. sativa*, *M. micrantha* and *V. vinifera* was performed. A total of 50,076 gene families were identified, of which 6,416 were shared by all 7 species, of which 5,251 were *A. argyi*-specific (**Figure 3A**). *A. argyi* had much more specific gene families than the other 6 plant species, 1,191 specific gene families in *A. annua*, 816 in *C. canephora*, 1,424 in *C. nankingense*, 821 in *L. sativa*, 2,006 in *M. micrantha* and 1,052 in *V. vinifera*, respectively (**Figure 3A**).

Furthermore, 31,073 *A. argyi*-specific genes were identified (**Supplementary Table 16**), which are annotated in **Supplementary Table 17**. A total of 18,889 and 8,262 *A. argyi*-specific genes were annotated to GO terms (**Supplementary Table 18**) and KEGG pathways (**Supplementary Table 19**), respectively. The GO annotations were mainly related to transposition, DNA-mediated, recombinational repair and actin filament depolymerization in the “biological process” term; extracellular space, actin cytoskeleton and nucleolus in the “cellular component” term; second spliceosomal transesterification activity, transposase activity and pre-mRNA 3’-splice site binding in the “molecular function” term (**Supplementary Figure 3**). The KEGG pathway analysis showed that “arginine and proline metabolism” and “linoleie acid metabolism” were the most significant (**Supplementary Figure 4**).

Gene family copy number analysis showed that the *A. argyi* gene family ranges from one to more than four copies, the result showed that *A. argyi* has much more genes in families with four or more members than the other 6 plant species (**Figure 3B**).

Further analysis of gene family expansion and contraction revealed that 6,607 gene families expanded and 430 gene families contracted in the *A. argyi* genome (**Supplementary Table 20**), after divergence from *A. annua* respectively (**Figure 3C**), suggesting that much more *A. argyi* gene families have experienced expansion than contraction during adaptive evolution. A total of 36,580 and 16,269 genes in the expanded genes were annotated to GO terms and KEGG pathways (**Supplementary Tables 21**, **22**), respectively. The GO annotations were mainly related to recognition of pollen, response to water and nitrate assimilation in the “biological process” term; U4 snRNP, spliceosomal tri-SNP complex and COPI vesicle membrane in the “cellular component” term; and chitin binding, ligand-gated ion channel activity and dioxygenase activity in the “molecular function” term (**Supplementary Figure 5**). The KEGG pathway analysis showed that 16269 of the genes from expanded gene families were clustered in 90 KEGG pathways, of which “zeatin biosynthesis”, “cutin”, and “suberine and wax biosynthesis” demonstrated the largest gene family expansion (**Supplementary Figure 6**). These 430 contract gene families were then annotated to GO terms and KEGG pathways (**Supplementary Tables 23**, **24**), respectively. The GO terms of the genes from contracted gene families were mainly related to nucleosome assembly and carboxylic acid metabolic in the “biological process” term; nucleosome and caveola in the “cellular component” term; and protein heterodimerization activity, carboxy-lyase activity and pyridoxal phosphate activity in the “molecular function” term (**Supplementary Figure 7**). KEGG analysis showed that most of the contracted genes were involving in histidine metabolism (**Supplementary Figure 8**).

Positive selection analysis identified 105 genes containing significantly positively selected sites. A total of 89 and 41 genes in the positive selection genes were also annotated (**Supplementary Table 25**) to GO terms and KEGG pathways (**Supplementary Tables 26**, **27**), respectively. GO analysis revealed that most of the expanded orthogroups were involved in regulation of mitotic cell cycle phase transition and trichome branching in the “biological process” term, plant epidermis development, chromosome in the “cellular component” term; metalloendopeptidase activity in the “molecular function” term (**Supplementary Figure 9**). 41 of the genes from positive selection gene families were clustered in 19 KEGG pathways, involved in inositol phosphate metabolism, and phosphatidylinositol signaling system (**Supplementary Figure 10**).

An analysis was performed with 6 other sequenced plant genomes, and 383 single-copy genes were used for phylogenetic tree construction. As expected, the results showed that *A. argyi* is relatively closely related to *A. annua* and *C. nankingense* (**Figure 3D**). The phylogenetic tree also indicated that *A. argyi* diverged phylogenetically from *A. annua* approximately 2–8 million years ago (Mya), after the divergence of *C. nankingense* at 3–10 Mya (**Figure 3D**).

### Dynamic changes of transcriptome after exogenous MeJA spraying

**Figure 4:**
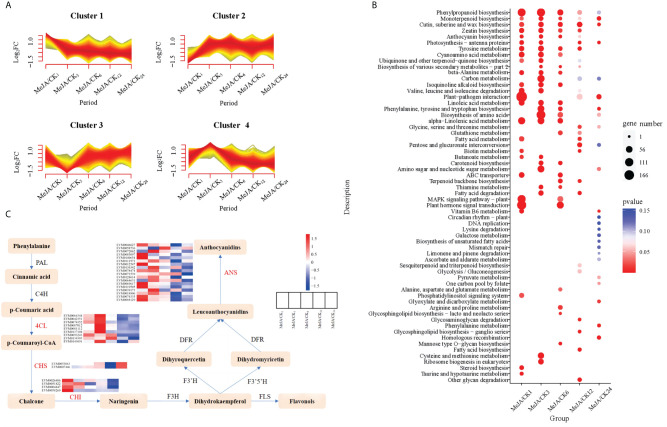
Multivariate statistical analysis of transcriptome data in *A. argyi* leaves treated by MeJA. **(A)** K-means clustering analysis of differentially expressed genes. **(B)** KEGG functional enrichment analysis of differentially expressed genes. **(C)** Pathway heatmap of key DEGs in significantly enriched biosynthetic pathways of phenylpropanoid, flavonoid, and anthocyanin.

MeJA, acting as an inducer, is an endogenous growth hormone crucial for regulating plant secondary metabolism (Wang et al., 2023). The exogenous application of MeJA can stimulate plants to synthesize secondary metabolites such as alkaloids, terpenoids, phenolics, anthocyanins, and volatile compounds (Chen et al., 2006). To explore the internal changes in *A. argyi* following exogenous MeJA treatment, we employed RNA-Seq to sequence *A. argyi* leaves treated with 100μM MeJA. The analysis unveiled genes with differential expression in response to MeJA treatment, shedding light on the key biological pathways influenced by MeJA. We constructed 30 cDNA libraries from leaves collected at 1, 3, 6, 12, and 24 hours post-treatment with each group biologically replicated three times. After sequencing quality control, low-quality reads were filtered out, resulting in a total of 195.02 Gb of clean reads, with the percentage of Q30 bases in each sample not less than 92.87% (**Supplementary Table 28**). The clean reads were aligned to the reference genome, and gene expression information was obtained, with the mapping efficiency of reads to the reference genome ranging between 83.95% and 84.52%. Using a threshold of Fold Change ≥ 1.5 and FDR < 0.05, differentially expressed genes (DEGs) at various MeJA treatment times were statistically analyzed (**Figure 4**; **Supplementary Figure 11**). The results revealed significant gene expression changes within the initial 6 hours following MeJA treatment (**Supplementary Figure 11**). To explore the expression patterns of DEGs at different time points, K-Means clustering was performed, integrating all DEGs into four major categories (**Figure 4A**). We observed that gene expression differences were more significant within the initial 6 hours after MeJA treatment, with gene expression changes stabilizing thereafter. By comparing the trends of DEGs, we speculated that *A. argyi* exhibited the most active gene expression within 6 hours after MeJA treatment in response to MeJA stress.

All DEGs detected in at least one comparison group were mapped to metabolic pathways in KEGG, with a focus on the top 25 pathways. KEGG pathway enrichment analysis revealed significant participation of DEGs in pathways such as phenylpropanoid biosynthesis, monoterpenoid biosynthesis, cutin/suberine and wax biosynthesis, zeatin biosynthesis, and anthocyanin biosynthesis, with notable enrichment in the initial three stages (**Figure 4B**). Previous research has indicated that the exogenous application of MeJA induced the biosynthesis of various secondary metabolites, including terpenoids, flavonoids, alkaloids, anthocyanins, phenylpropanoids, and glucosinolates (Chen et al., 2006). By integrating KEGG enrichment results with gene function annotations, critical DEGs involved in the biosynthesis pathways of phenylpropanoids and flavonoids were identified, including flavonoid biosynthesis (ko00941), phenylpropanoid biosynthesis (ko00940), and anthocyanin biosynthesis (ko00942). A total of 33 key DEGs were identified, involving enzymes such as *4-coumarate-CoA ligase* (*4CL*), *chalcone synthase* (*CHS*), *chalcone isomerase* (*CHI*), and *anthocyanidin synthase* (*ANS*) (**Supplementary Table 29**). Similarly, the differential expression of these key genes was primarily concentrated within the initial 6 hours post-MeJA treatment (**Figure 4C**). Furthermore, it was observed that during the initial three stages, up-regulated DEGs included a subset of *caffeic acid 3-O-methyltransferase* (*COMT*) associated with lignin synthesis (**Supplementary Figure 12**). Overall, the expression of these genes exhibited an initial increase followed by a decrease after exogenous MeJA application. This suggests an increase in lignin content in *A. argyi* following MeJA treatment, indicating a potential correlation with enhanced stress tolerance in plants post-exogenous MeJA application. Additionally, *COMT* catalyzes the direct conversion of caffeic acid to ferulic acid, an important precursor in ferulic acid biosynthesis (Gowri et al., 1991). Therefore, COMT is considered one of the key enzymes involved in ferulic acid biosynthesis. Modern research has demonstrated that ferulic acid and its derivatives possess various beneficial effects such as neuroprotection, analgesia, antioxidant activity, anti-thrombotic properties, and anticancer activity (Zduńska et al., 2018; Liu et al., 2022). The identification of these genes lays the foundation for breeding new varieties of *Artemisia* with higher concentrations of ferulic acid.

### Regulation of MeJA on terpenoid biosynthesis

**Figure 5:**
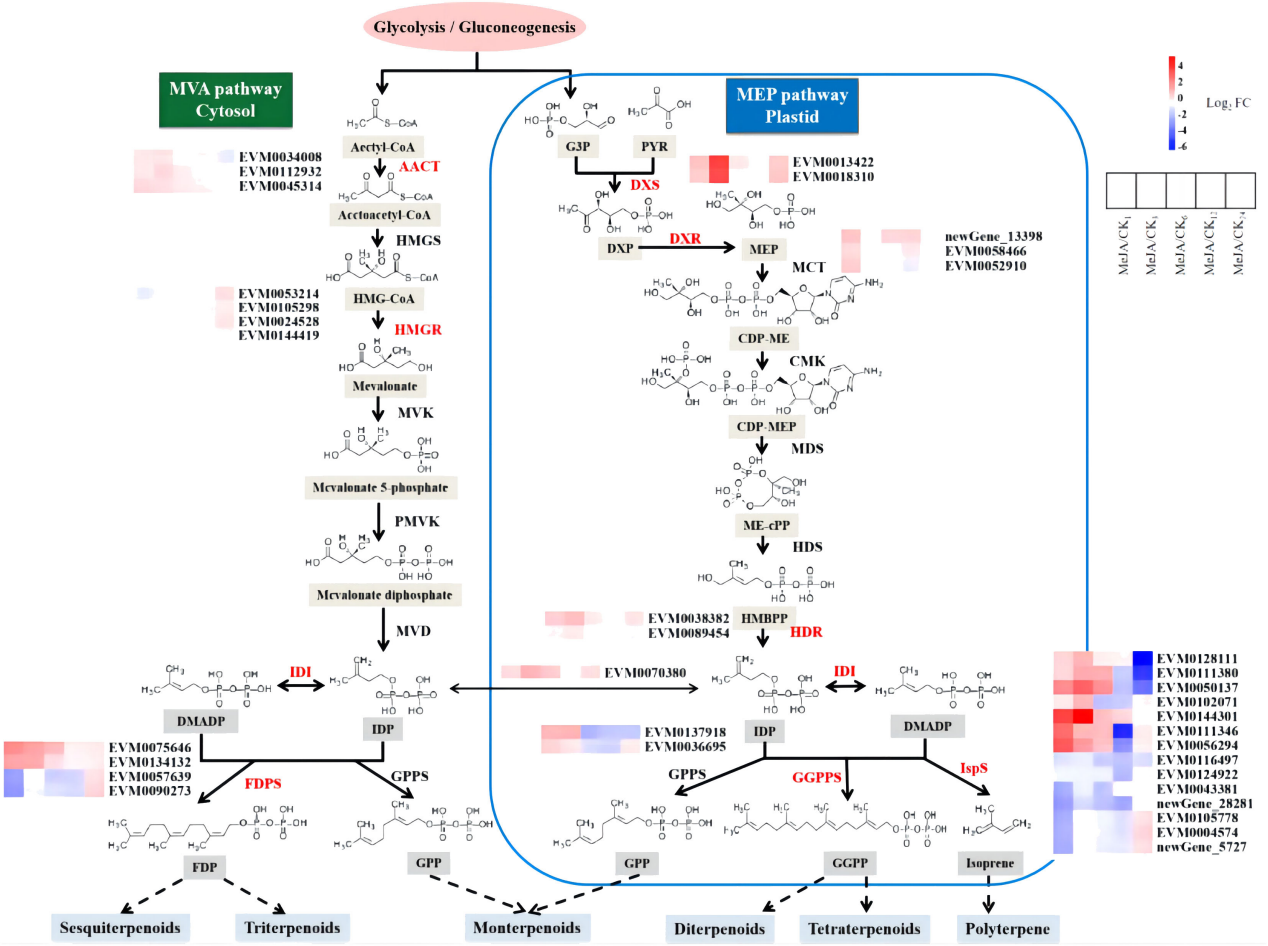
DEGs involved in terpenoids biosynthesis in *A. argyi* leaves induced by MeJA. The significantly differentially expressed enzymes were labeled with color, in which the up-regulated ones were red and the down regulated ones were blue. AACT, Acetyl-CoA C-acetyltransferase; HMGS, hydroxymethylglutaryl-CoA synthase; HMGR, hydroxymethylglutaryl-CoA reductase (NADPH); MVK, mevalonate kinase; PMVK, phosphomevalonate kinase; MVD, diphosphomevalonate decarboxylase; DXS, 1-deoxy-D-xylulose-5-phosphate synthase; DXR, 1-deoxy-Dxylulose-5-phosphate reductoisomerase; MCT, 2-C-methyl-D-erythritol 4-phosphate cytidylyltransferase; CMK, 4-diphosphocytidyl-2-C-methylD-erythritol kinase; MDS, 2-C-methyl-D-erythritol 2,4-cyclodiphosphate synthase; HDS, (E)-4-hydroxy-3-methylbut-2-enyl-diphosphate synthase; HDR, 4-hydroxy-3-methylbut-2-en-1-yl diphosphate reductase; IDI, isopentenyl-diphosphate Delta-isomerase; DMADP, dimethylallyl diphosphate; GPPS, geranyl diphosphate synthase; FPPS, farnesyl diphosphate synthase; GGPPS, geranylgeranyl diphosphate synthase; IspS, Isoprene synthase.

Terpenoids in *A. argyi* constitute an important and valued component (Yoshikawa et al., 1996). Among the expanded gene families specific to *A. argyi* and its unique genes, those involved in terpenoid backbone biosynthesis (ko00900) and monoterpenoid biosynthesis (ko00902) are notably enriched (**Supplementary Table 19**). Besides, one of the 41 gene families under positive selection is also involved in terpenoid backbone biosynthesis (ko00900), suggesting potential roles related to terpenoid production (**Supplementary Table 27**). The main components of volatile oil from *A. argyi* leaves are terpenoids. To elucidate the regulation mechanism of MeJA on terpenoid biosynthesis in A. argyi, we investigated the differential expression of structural genes in the terpenoid backbone biosynthesis pathway in response to MeJA treatment (**Figure 5**; **Supplementary Table 30**). The results revealed that MeJA slightly promoted the expression of *AACT*, the first step catalytic enzyme of MVA pathway. Similarly, their primary roles were concentrated in the initial three stages. In contrast, one of the most critical rate-limiting enzymes in the MVA pathway, *HGMR*, did not show significant differences in gene expression during the initial 6 hours post-treatment, with expression levels rising after 6 hours. Regarding *FDPS*, both *EVM0075646* and *EVM0134132* exhibited sustained higher expression levels than the control within 24 hours after MeJA treatment. Conversely, *EVM0057639* and *EVM0090273* only showed preferential expression 24 hours post-treatment. Therefore, MeJA may partially enhance the terpenoid biosynthesis of the MVA pathway in A. argyi. Additionally, a systemic up-regulation of key genes by exogenous MeJA in MEP pathway, including genes encoding the major rate-determining enzymes (*DXS* and *HDR*, the first and the last enzyme of the MEP pathway) and another potential control point (*DXR*) of the metabolic flux to plastidial isoprenoids. Furthermore, the increased expression levels of *IDI* in terpenoid biosynthesis pathway possibly led to increased terpenoid production in MeJA-treated *A. argyi*. Interestingly, the expression of GPPS did not respond to MeJA, indicating that MeJA may not induce monoterpene synthesis in *A. argyi*. Similar to *FDPS*, half of the *IspS* genes were up-regulated by MeJA within the first 6 hours, while the other half showed up-regulation between 12–24 hours, suggesting that MeJA may regulate the biosynthesis of sesquiterpenes, triterpenes, and polyterpenes differently.

## Discussion

*A. argyi* holds significant importance in Chinese traditional medicine due to its wide-ranging pharmacological effects, highly esteemed by consumers. The *de novo* assembly of large genomes with a high degree repeat content remains a challenge (Claros et al., 2012; Kajitani et al., 2014). However, sequencing platforms such as the Oxford Nanopore, PacBio RS II and Sequel systems are designed to generate long reads, which greatly facilitates the sequence assembly process and allows high-quality assemblies to be generated. Here, using Nanopore technology, the genome sequence of *A. argyi* was assembled, which provides a basic resource for the further exploration of the growth characteristics, cultivation and molecular breeding of *A. argyi*. A genome sequence of 4.15 Gb was assembled with a contig N50 of 0.51 M (**Table 1**). However, these obtained sequences were not assembled into chromosomes, we will assemble these obtained sequences to chromosome-level by Hi-C in future. The quality of the assembly was further assessed by three independent methods. Thus, the obtained *A. argyi* genome not only provides basic information for research and utilization, but also holds promise for enhancing our understanding genome evolution and plant relationships within the Asteraceae family.

A total of 147,248 genes were predicted in the *A. argyi* genome, surpassing the gene count in other plant species such as *A. annua* (N = 63,226), *C. nankingense* (N = 56,870) and *L. sativa* (N = 3,5382) (**Supplementary Table 10**). We identified an abundance of repetitive elements in the *A. argyi* genome (68.86% of the assembly), among which the LTR retrotransposons (Gypsy and Copia) were the most abundant (21.37% and 12.27%, respectively, **Supplementary Table 14**). A high content of repetitive elements is a shared feature among several known large genomes of the Asteraceae family. For instance, in *Helianthus annuus* L., more than 75% of the genome consists of LTRs, with a relatively high proportion (59%) being Gypsy repeats (Badouin et al., 2017). In *L. sativa*, 74.2% of the genome is occupied by repetitive regions, with the LTR/Gypsy subfamilies (33.9%) being the most abundant (Reyes-Chin-Wo et al., 2017). In *A. annua*, 61.56% of the genome is repetitive elements and the most abundant characterized elements were LTRs (22.69%) (Qian et al., 2018). In *C. nankingense*, 69.6% of the genome is identified as repetitive elements, among which the LTR retrotransposons (Copia and Gypsy) were the most abundant (46.9%) (Song et al., 2018). We found recent LTR retrotransposon bursts in the *A. argyi* genome (**Supplementary Figure 2**). In the genomes of tea, *C. nankingense*, and *Picea abies*, long-lasting and uninterrupted LTR retrotransposon bursts may have led to extreme increases in genome size due to the lack of efficient DNA removal mechanisms (Nystedt et al., 2013; Xia et al., 2017; Song et al., 2018). Therefore, the recent LTR retrotransposon bursts in the *A. argyi* genome might have also significantly contributed to genome size. These findings underscore the critical role of transposable elements in the evolution of large plant genomes.

The KEGG and GO analyses of *A. argyi*-specific genes, expanded genes and positive selection genes revealed their involvement in terpenoid, phenylpropanoid, flavonoid, phenylalanine, polysaccharide and anthocyanin biosynthetic or metabolic process. All these pathways stem from the shikimate pathway, with terpenoids, flavonoids, polysaccharides, and anthocyanins being the primary active compounds. The classical flavonoid synthesis pathway starts from phenylalanine, which undergoes metabolism through the shikimate pathway. Phenylpropanoids are considered as key mediators in plant resistance to biotic and abiotic stress responses (Vogt, 2010).

The phylogenetic tree constructed from the whole genome analyses showed that *A. argyi* diverged from *A. annua* approximately 2–8 Mya. The estimated times of the separation from the lineages with *C. canephora* and *V. vinifera* were approximately 93–107 and 110–124 Mya, respectively.

The exogenous application of MeJA can stimulate plants to synthesize secondary metabolites such as alkaloids, terpenoids, phenolics, anthocyanins, and volatile compounds (Chen et al., 2006). In this study, we verified this concept through exogenous MeJA application and transcriptome sequencing results. Our findings revealed that DEGs was involved in phenylpropanoid biosynthesis, monoterpenoid biosynthesis, cutin/suberine and wax biosynthesis, zeatin biosynthesis, and anthocyanin biosynthesis. Furthermore, we observed that the initial 6 hours after exogenous MeJA application exerted the most significant effect on the physiological process of *A. argyi*. MeJA exogenous treatment may regulate the biosynthesis of sesquiterpenes, triterpenes, and polyterpenes differently. It can rapidly increase the expression of MEP and MVA pathway-related genes within a short timeframe, with evident responses observed within 12 hours. Therefore, through biotechnology, altering the expression levels of related target genes or editing the target genes in *A. argyi*, we can develop genetically improved individual plants with significantly increased yield of secondary metabolites such as terpenoids. Moreover, it is imperative to explore the transcriptional regulatory factors responsive to MeJA to elucidate the molecular mechanisms underlying MeJA’s promotion of terpenoid synthesis and to construct the regulatory network of secondary metabolite biosynthesis in *A. argyi*. Aside from plant hormones, other environmental factors can also influence the content and yield of secondary metabolites, including light, low temperature, salt, drought, heavy metals and diseases and insect pests (Isah, 2019). Therefore, it is crucial to consider the regulation of these environmental factors on the growth and development of *A. argyi*, as well as the interactions between environmental factors and *A. argyi* plants.

In summary, this is a report for sequencing, assembly, annotation and comparative genomics analyses of *A. argyi* genome, an important medicinal plant with widespread applications worldwide. We have further identified and enriched candidate genes associated with the phenylpropanoid, flavonoid and terpenoid biosynthesis pathways, which produce active compounds in this medicinal plant, and verified the regulatory effect of MeJA on these pathways. The findings of our study will provide valuable insights for furthering fundamental biological research and applied breeding programs, aiming to enhance the effective utilization of *A. argyi*.

## Data availability statement

The data presented in the study are deposited in the NCBI Sequence Read Archive (SRA) database and GenBank repository, accession number PRJNA747845, PRJNA794198 and JAKLPF000000000.

## Author contributions

XG: Writing – original draft, Writing – review & editing. QM: Data curation, Methodology, Writing – review & editing. XZ: Data curation, Writing – review & editing, Visualization. XW: Investigation, Writing – review & editing. NW: Visualization, Writing – review & editing. YC: Writing – review & editing. SL: Writing – review & editing. SM: Writing – review & editing. HW: Writing – review & editing. KZ: Formal analysis, Funding acquisition, Resources, Supervision, Writing – review & editing.
