# Supplementary figures and images for: The reference genome sequence of Artemisia argyi provides insights into secondary metabolism biosynthesis
Source: Front Plant Sci. 2024 Jun 28;15:1406592. doi: 10.3389/fpls.2024.1406592 (PMC11239399; doi:10.3389/fpls.2024.1406592)

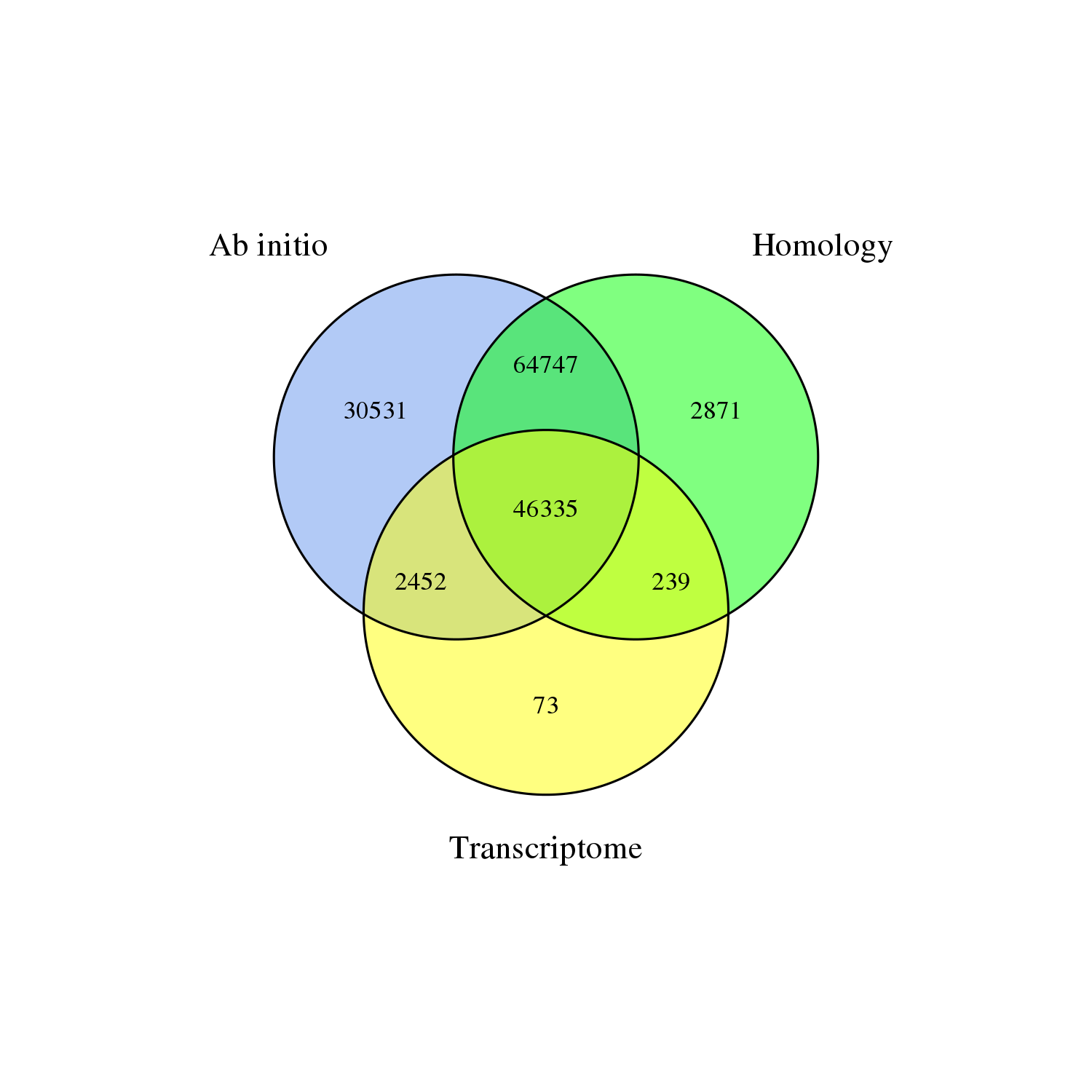

Supplement: Supplementary file 2 [file Image_1.png]

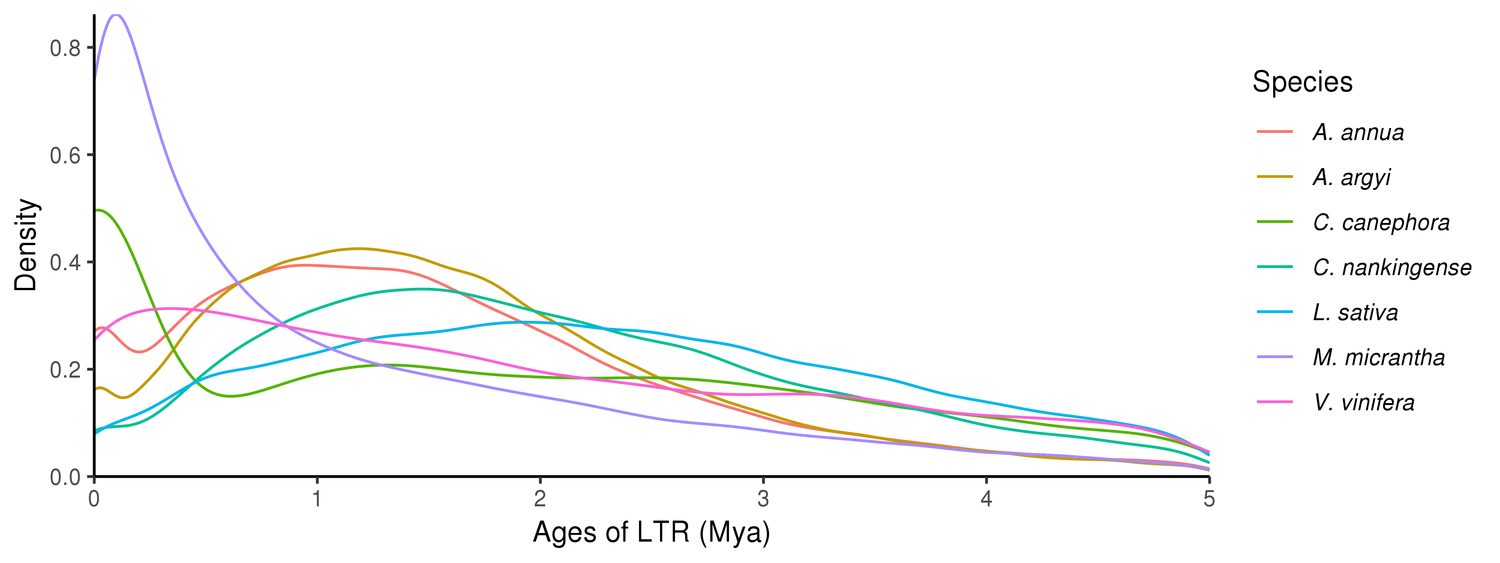

Supplement: Supplementary file 3 [file Image_2.png]

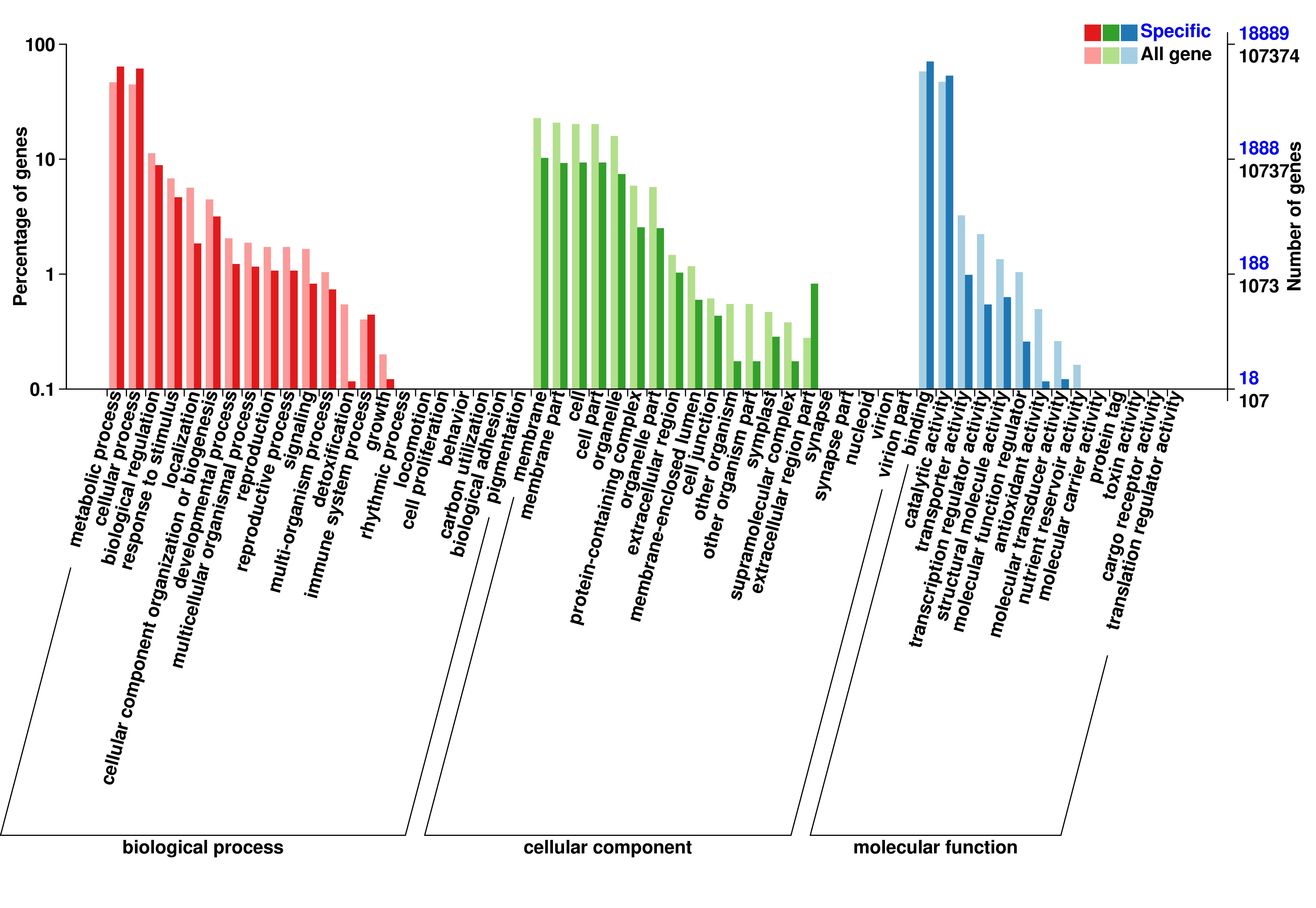

Supplement: Supplementary file 4 [file Image_3.png]

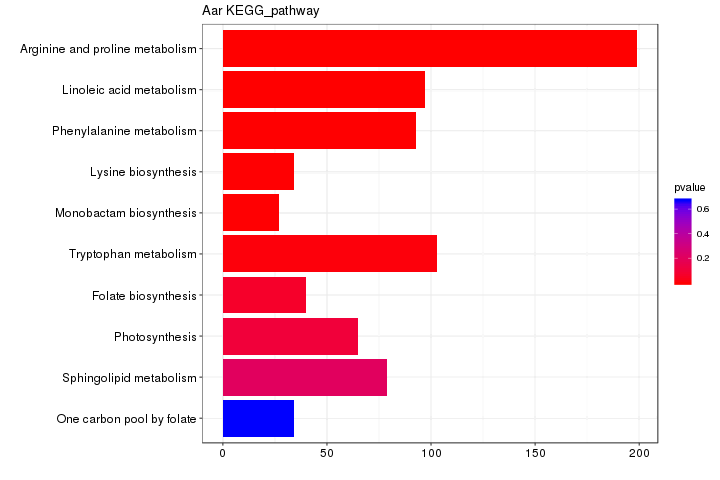

Supplement: Supplementary file 5 [file Image_4.png]

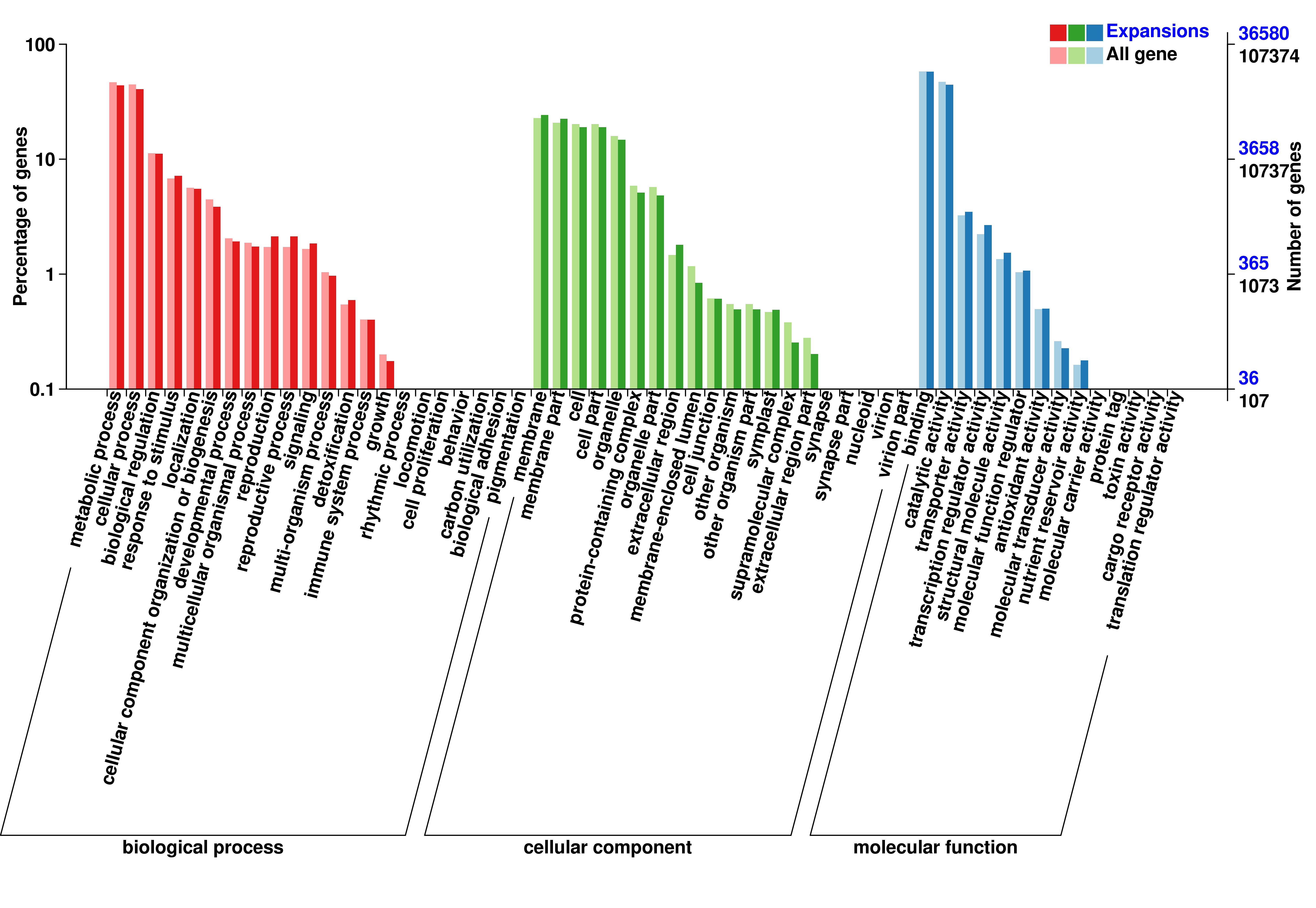

Supplement: Supplementary file 6 [file Image_5.png]

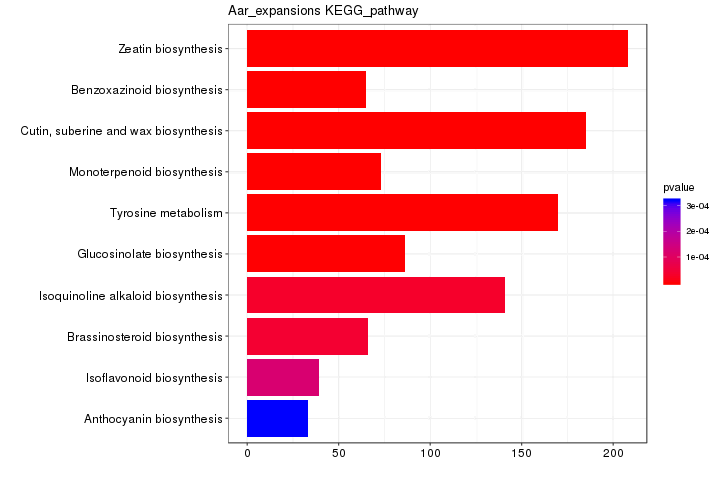

Supplement: Supplementary file 7 [file Image_6.png]

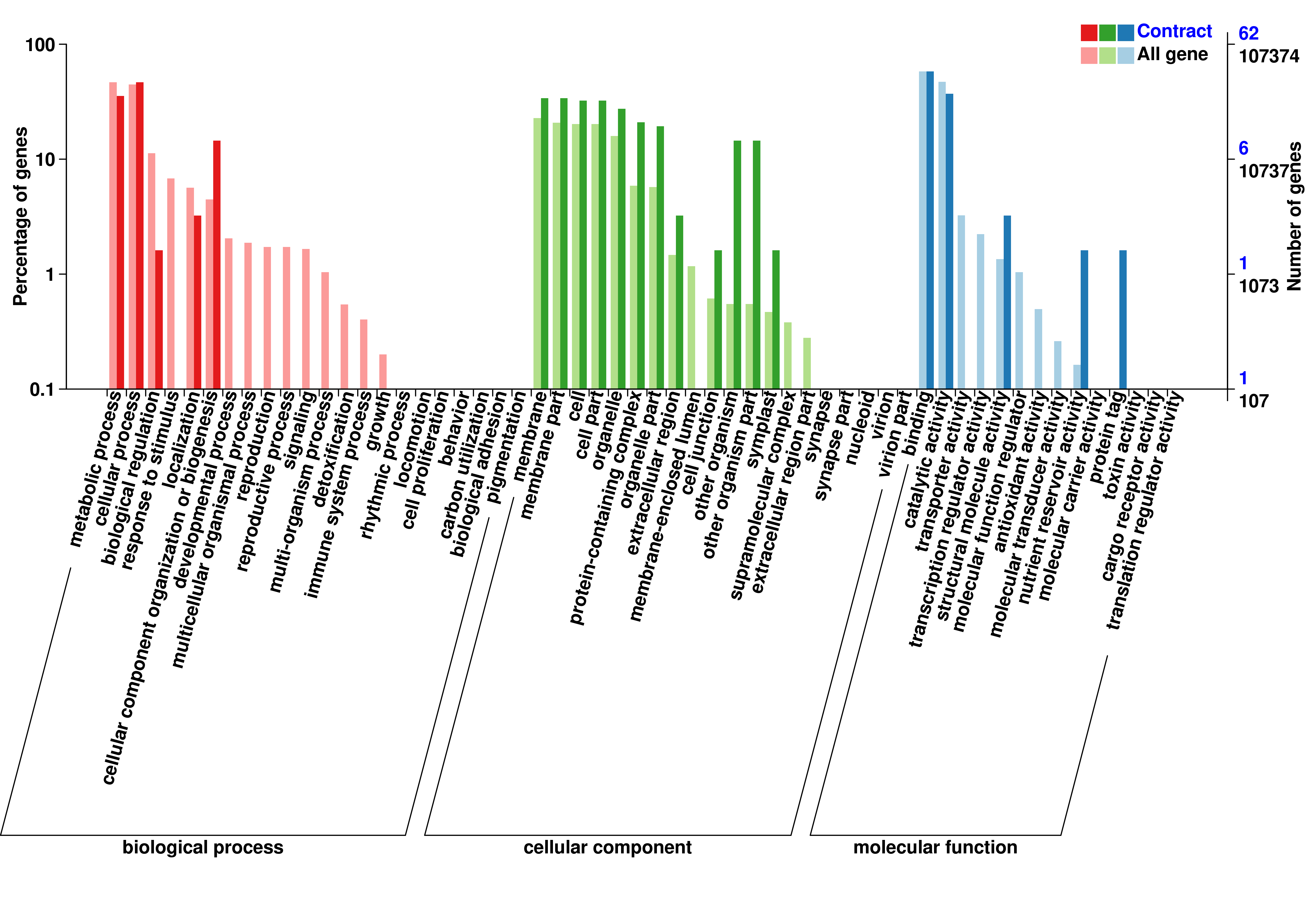

Supplement: Supplementary file 8 [file Image_7.png]

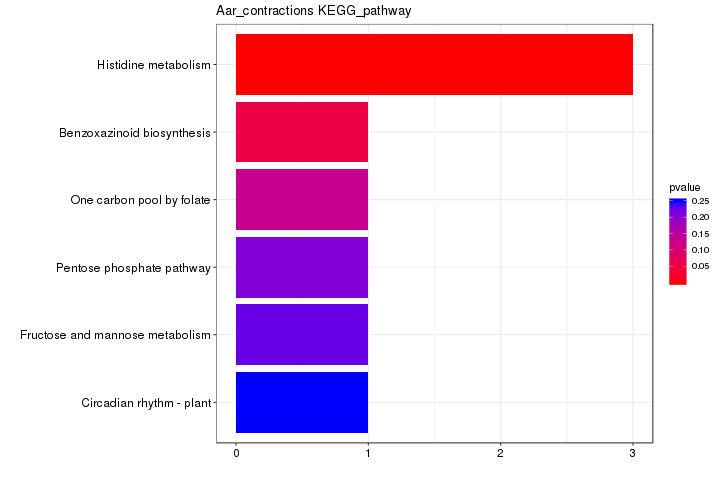

Supplement: Supplementary file 9 [file Image_8.png]

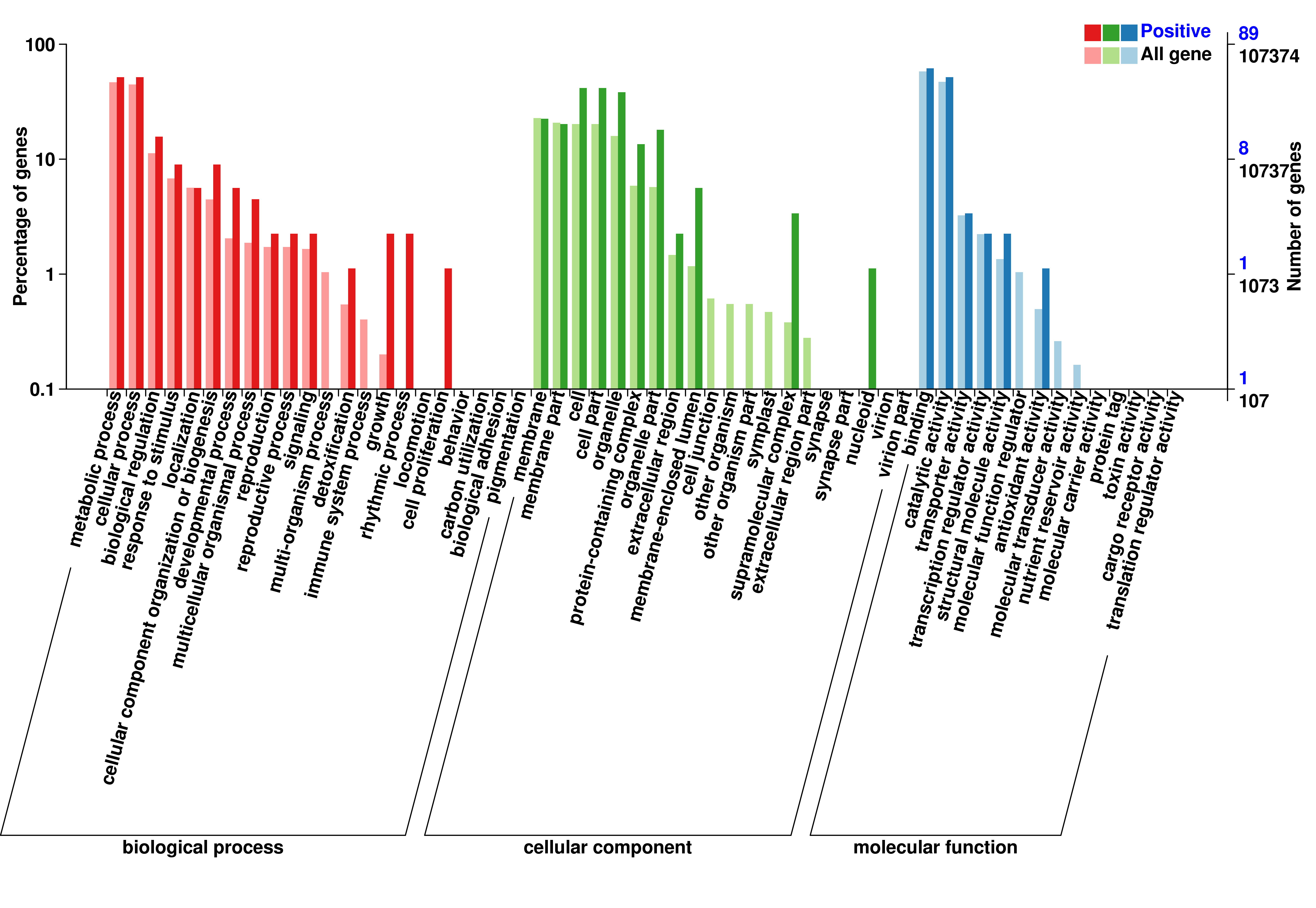

Supplement: Supplementary file 10 [file Image_9.png]

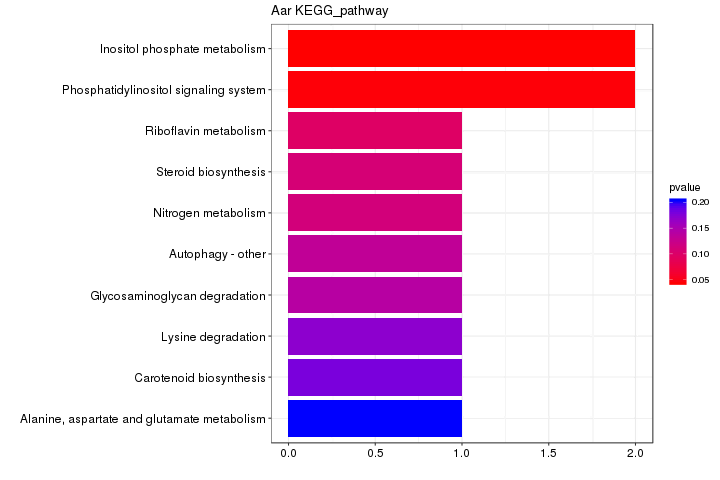

Supplement: Supplementary file 11 [file Image_10.png]

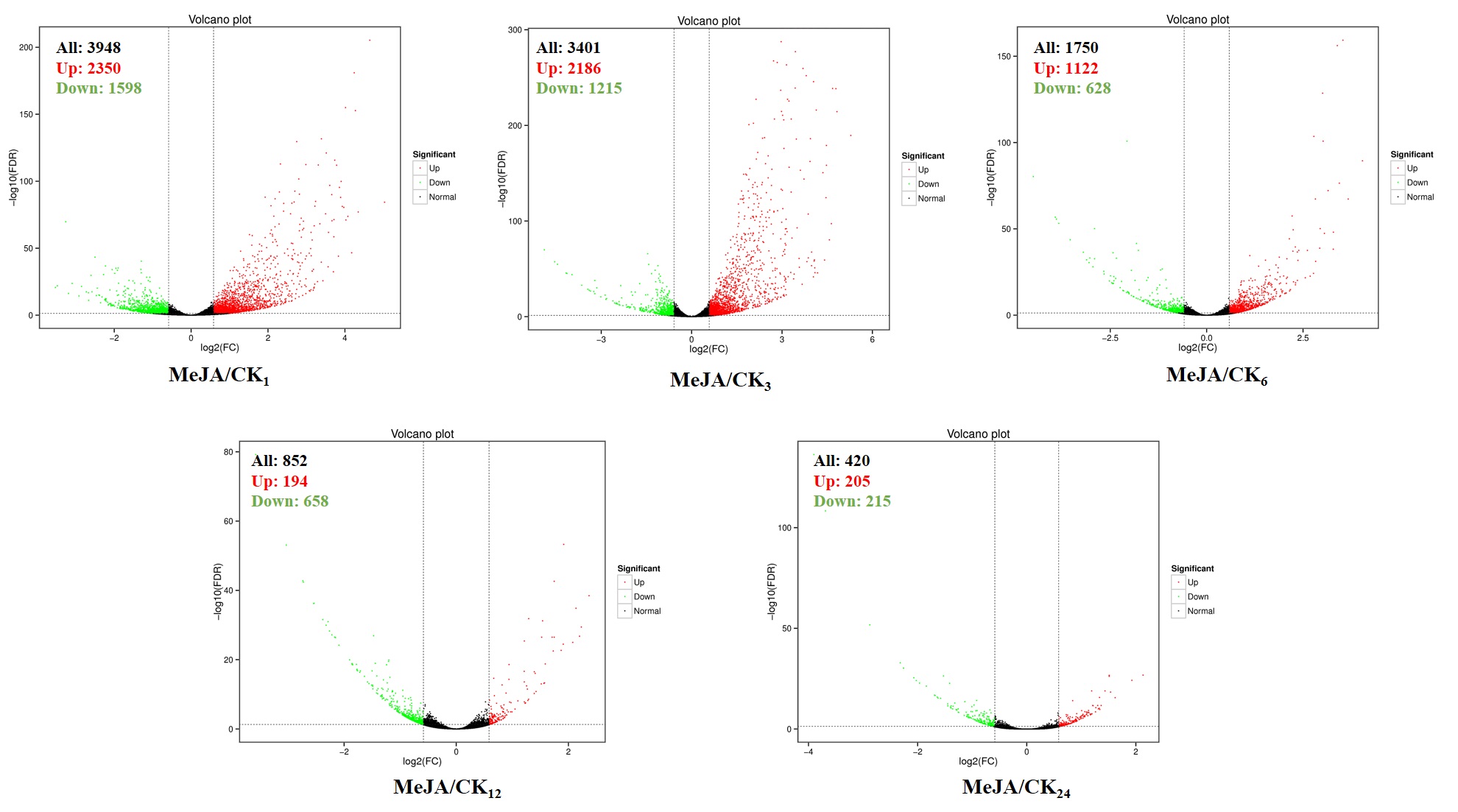

Supplement: Supplementary file 12 [file Image_11.jpeg]

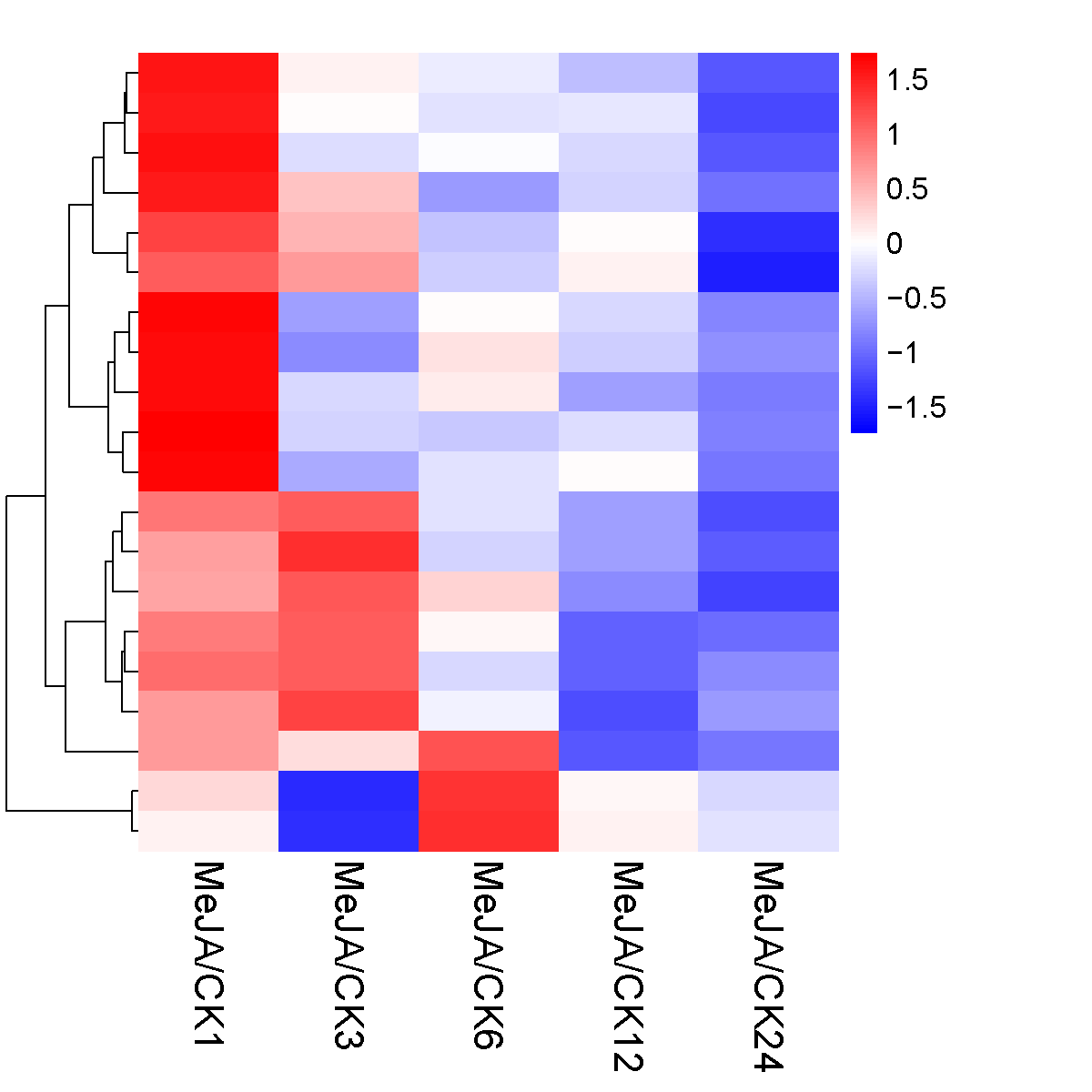

Supplement: Supplementary file 13 [file Image_12.png]
